# Restoration of Non-Uniform Motion-Blurred Star Images Based on Dynamic Strip Attention

**DOI:** 10.3390/jimaging12030103

**Published:** 2026-02-27

**Authors:** Jixin Han, Zhaodong Niu, Jun He

**Affiliations:** National Key Laboratory of Science and Technology on ATR, College of Electronic Science and Technology, National University of Defense Technology, Changsha 410073, China; hanjixin23@nudt.edu.cn (J.H.); jhe@nudt.edu.cn (J.H.)

**Keywords:** space objects, image deblurring, variable attention mechanism, deep learning, star image

## Abstract

When capturing star images in long-exposure mode, due to the relative motion between stars and space objects and the observation camera, strip tailings with different directions and lengths will be formed, resulting in a serious decline in image quality and inaccurate centroid positioning. Traditional methods for restoring star images are prone to ringing effects and cannot restore the non-uniformly blurred star images. Aiming at this problem, this paper proposes a star image restoration network based on a dynamic strip attention mechanism. Firstly, a Multi-scale Dynamic Strip Pooling Module is designed to adaptively extract blurred features of different lengths and directions by dynamically adjusting the strip convolution. After that, a Multi-scale Feature Fusion Module is designed to fuse multi-level features to reduce the loss of image details of stars and space objects in the image. Experimental results demonstrate that the proposed method achieves a PSNR of 84.08 and an SSIM of 0.9928 on the 16-bit simulated dataset, outperforming both traditional methods and other deep learning-based approaches. Specifically, the recognition accuracy of star points is increased by 174% in comparison with unprocessed images. Furthermore, this paper validates the network using the real-world dataset spotGEO, and the results indicate that the average number of successfully recognized star points is increased by 57% compared to direct processing of the original images.

## 1. Introduction

With the development of space programs worldwide, human beings have progressively expanded their activities into the field of space. The increasing number of satellites and spacecraft launched into orbit has exacerbated the problem of space debris. These uncontrolled space debris [1] in deep space pose a threat to the normal operation of satellites in orbit, making the cataloging and detection of such debris critically important.

**Figure 1:**
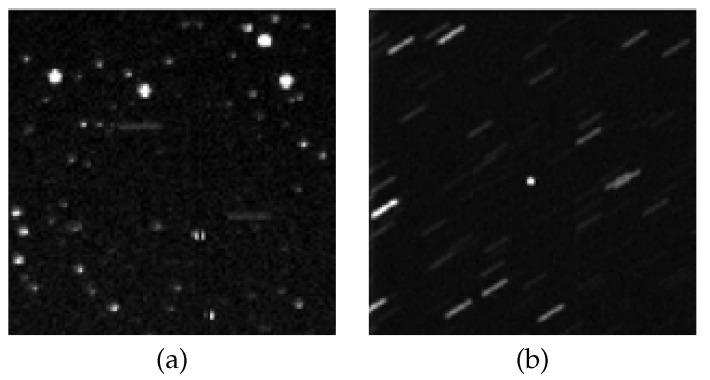
Schematic diagram of different observation modes: (**a**) Stellar-gazing mode, (**b**) Target-tracking mode.

The use of optical telescopes to observe space objects offers advantages such as low operating costs, long detection range, high measurement accuracy, and strong concealment [2]. The operational modes of optical telescopes can be categorized into two types: the first is the ‘stellar-gazing mode’, in which the telescope maintains a fixed gaze on a stationary star; the second is the ‘target-tracking mode’, in which the telescope requires continual readjustment to sustain its gaze on a spatial target. The specific differences between these two modes are illustrated in Figure 1:

When optical telescopes employ long exposure times across different observation modes, they accumulate signals from star points. However, relative motion between these star points and the detector’s imaging plane causes elongated “trailing” effects in the star images [3]. This disperses the star point energy across more pixels, resulting in blurred or fragmented point representations. Consequently, the accuracy of star centroid extraction is compromised, hindering precise star image recognition in subsequent processes.

Image deblurring can be generally categorized into traditional methods and deep learning-based methods. Traditional methods typically infer the relationship between the blur kernel and the sharp image in an inverse manner via deconvolution algorithms. Depending on whether the blur kernel is known, they can be further divided into non-blind and blind image deblurring methods. Non-blind image deblurring methods [4,5,6] solve for the underlying sharp image using deconvolution techniques when the blur kernel is known. In contrast, blind image deblurring methods [7,8,9,10,11] first estimate the blur kernel through prior modeling approaches when it is unknown, then derive the solution. Common methods for blurred image restoration include inverse filtering [12], Wiener filtering [13], regularized filtering [14], and Lucy-Richardson filtering (L-R) [15,16]. Deep learning-based methods [17,18,19,20,21,22] do not require manual preconfiguration of various image priors and do not need to estimate the blur kernel. Leveraging the powerful feature extraction capability of convolutional neural networks, they establish a mapping from blurred images to sharp images, enabling an end-to-end image deblurring process.

Under long exposures or in high-dynamic-range conditions, star points in images exhibit trailing phenomena, i.e., motion blur [23]. Numerous scholars have investigated both traditional methods and deep learning approaches. Jiang et al. [24] proposed an improved Radon transform combined with a Z-function and a dual-threshold mask. They estimated the blur kernel from a single image and achieved restoration using an improved RL acceleration algorithm. However, the Radon transform is sensitive to noise and image resolution, which can easily lead to errors in blur kernel angle estimation. Hou et al. [25] quickly estimated the motion blur angle via the bispectral transform and PCA, estimated the blur length by combining the Radon transform with adjustable weights, and finally reconstructed the star points using a regional filtering method based on the super-Laplacian prior restoration algorithm. Nevertheless, this method relies on empirically set thresholds, resulting in poor stability. Additionally, some scholars have estimated the blur kernel using gyroscope data [26,27,28], but this approach depends on assumptions about the motion model. Zhang et al. [29] proposed a simple and effective $l_{p}\left(0<p\leq 1\right)$ regularization deblurring method based on star image brightness, which is applicable only to uniform blur scenarios in star images.

Chen et al. [30] proposed a deep learning method for star image motion blur, estimating blur kernel parameters essential for star deblurring, including blur angle and blur length, using sparse representation, a super-Laplacian prior, and an integrated neural network. To improve star sensor performance in high-dynamic-range environments, Liu et al. [31] proposed a Richardson-Lucy (RL) algorithm based on a radial basis function neural network (RBFNN). However, both methods require auxiliary processing and do not achieve end-to-end direct restoration. Zhang et al. [32] proposed an improved network based on MIMO-UNet, replacing the ResBlock module with the Res FFT-Conv module. This module learns image features from both the spatial and frequency domains, thereby enhancing the network’s deblurring performance. Wang et al. [33] incorporated motion flow maps and sharp star points as supervision signals, conducting end-to-end training via conditional generative adversarial networks (cGANs) to achieve direct restoration of motion-blurred star maps. However, both methods require large-scale datasets for network training and impose high demands on dataset quality.

Traditional methods for star image deblurring often require prior knowledge of the blur kernel, which is difficult to obtain in practical applications. Additionally, their restoration performance depends on the quality of the original image, making them prone to generating ringing artifacts [34]. When dealing with star images with complex backgrounds and non-uniform blur, traditional methods often exhibit limitations that restrict their effectiveness under variable observation conditions. However, the datasets used in current deep learning methods are often common ones, such as the GOPRO dataset [35] and RealBlur dataset [36], with no dedicated datasets for star images. On the other hand, the starfield detection images employed in this study present challenges in reconstructing overlapping regions where motion blur occurs between neighboring stars and space debris. Furthermore, the inconsistent motion directions of space debris relative to stars within the detection images result in multi-directional, multi-scale non-uniform blurring. This phenomenon complicates the simultaneous accurate reconstruction of blur kernels across different regions.

To address the issues above, this paper proposes a star image motion blur restoration network based on a variable attention mechanism. It adopts the Multi-scale Dynamic Strip Pooling Module (MDSPM) to extract image features and capture contextual blur information at different scales. Furthermore, the Multi-scale Feature Fusion Module (MFFM) fuses the extracted feature maps. Ultimately, the blurred star images are deblurred into sharp images, laying a foundation for subsequent star point detection.

## 2. Materials and Methods

### 2.1. Image Degradation Model

When capturing images with optical telescopes, spatial degradation occurs in the resulting space exploration images due to diffraction within the optical system, lens distortion of the sensor, relative motion between stars and the camera, and noise effects during imaging and transmission process. In modeling the star image degradation process, the optical imaging system is usually regarded as a linear time-invariant system [37]. When the blurring in an image is uniform, the blurring process can be represented by Equation (1), which is a simple convolution:

(1) $$I_{b}=h*I_{s}+n$$

where $I_{s}$ denotes the original sharp image, *h* represents the spatially uniform blurring kernel, ∗ is the convolution operation, $I_{b}$ stands for the blurred image, and n(x,y) represents noise. When the blurring kernel ceases to be uniform, the mathematical model may be expressed as a linear transformation [38]:

(2) $$I_{b}=H\cdot I_{s}+n$$

where *H* denotes the blur matrix, with each row corresponding to the blur kernel modeling at a specific pixel location.

For motion blur, its blur kernel function is given by Equation (3), where *L* denotes the motion blur length, θ represents the blur angle relative to the x-axis, and non-uniformly blurred images contain multiple h(x,y):

(3) $$h\left(x,y\right)=\left\{\begin{array}{cc} \frac{1}{L} & \mathrm{if}\;\sqrt{x^{2}+y^{2}}\leq \frac{L}{2},\;\theta =\arctan\left(\frac{y}{x}\right)\\ 0 & \mathrm{otherwise} \end{array}\right.$$

The application of the frequency domain transform to h(x,y) in Equation (3) results in the following frequency domain representation:

(4) $$H\left(u,v\right)=\frac{\sin\left[\pi L\left(u\cos\theta +v\sin\theta \right)\right]}{\pi L\left(u\cos\theta +v\sin\theta \right)}$$

### 2.2. Network Architecture of MSDeblurNet

**Figure 2:**
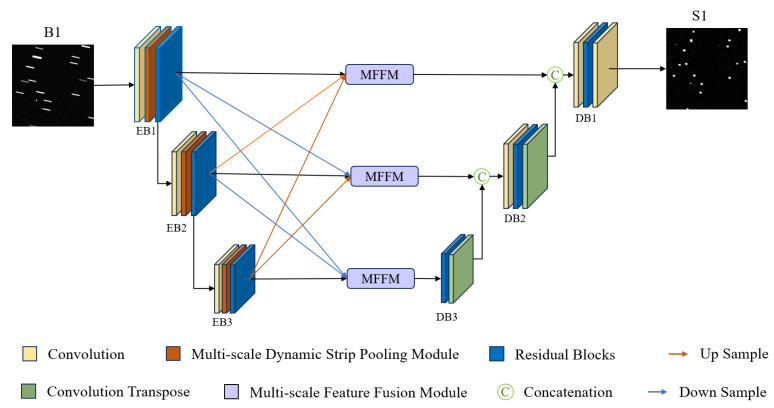
Network structure of MSDeblurNet.

The network architecture of MSDeblurNet (Multi-Scale DeblurNet) is illustrated in Figure 2. The network takes 16-bit single-channel star detection images of size 1024×1024 as input. These images exhibit blurring with overlapping motion blur regions and non-uniform motion blur (i.e., containing multiple blur kernels with varying angles and lengths). The network primarily comprises an encoder, a multi-scale feature fusion module, and a decoder.

The network first extracts features from the input images via the encoder. Each encoder layer comprises convolutional layers, a Multi-scale Dynamic Strip Pooling Module (MDSPM), and eight residual blocks. Specifically, the MDSPM is designed to extract multi-scale contextual information from the image, enabling the network to capture motion blur characteristics of diverse sizes and directions and thereby mitigating blurring artifacts in the input. Subsequently, the Multi-scale Feature Fusion Module fuses the multi-resolution feature maps extracted by the network, providing rich feature representations that facilitate the restoration of overlapping, occluded blur and non-uniform motion blur. The decoder employs eight residual blocks to reconstruct the output features, ultimately restoring motion-blurred star detection images and yielding high-quality, sharp detection images.

### 2.3. Multi-Scale Dynamic Strip Pooling Module

During long exposure of the telescope, relative motion between stars and space debris induces streak trails on the image plane. Debris moving in diverse directions gives rise to inconsistent blur kernels, which require separate estimation and thereby exacerbate the difficulty of restoration. Furthermore, when streaked stars overlap with each other, the edges of adjacent stars become blurred and merged, resulting in ambiguous edge information.

**Figure 3:**
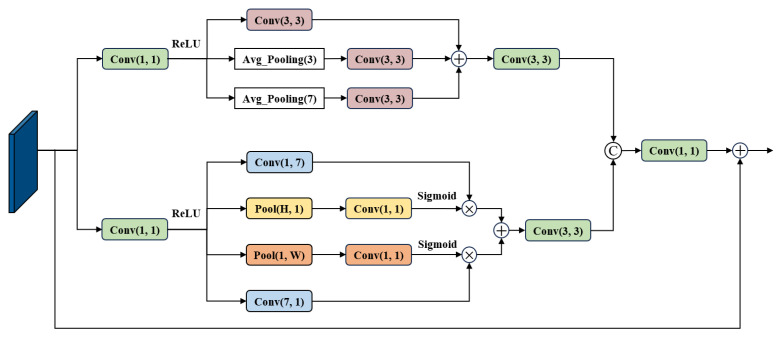
Block diagram of Multi-scale Dynamic Strip Pooling Module.

To address the above issues, this paper designs a Multi-scale Dynamic Strip Attention Mechanism Module. The Strip Attention Mechanism captures horizontal and vertical information in the image, adjusting spatial pixel weights to emphasize key features. Given the characteristics of non-uniform motion blur, square pooling at different scales and the Multi-scale Dynamic Strip Attention Mechanism are designed in the feature extraction stage. While preserving the key spatial information of the input feature maps, this module captures blur features at various scales in the image, followed by feature extraction and enhancement. The specific structure of the module is illustrated in Figure 3:

Let the module input be *X*. Through two parallel 1×1 convolutions, intermediate features $X_{1}$ and $X_{2}$ are generated, respectively, achieving dimensionality reduction across channels and preliminary feature extraction.

(5) $$\left\{\begin{array}{c} X_{\mathrm{pool}}={\mathrm{Conv}}_{3\times 3}\left(X_{1}\right)+{\mathrm{Conv}}_{3\times 3}\left({\mathrm{Pool}}_{3}\left(X_{1}\right)\right)+{\mathrm{Conv}}_{3\times 3}\left({\mathrm{Pool}}_{7}\left(X_{1}\right)\right)\\ X_{1}=\mathrm{ReLU}\left({\mathrm{Conv}}_{1\times 1}\left(X\right)\right) \end{array}\right.$$

Equation (5) represents the multi-scale square pooling branch, corresponding to the upper half of Figure 3. Three parallel processing streams extract local blurred features at different scales. The three feature streams are element-wise summed to yield $X_{\mathrm{pool}}$, thereby achieving adaptive perception capabilities for blurring at varying scales.

(6) $$\left\{\begin{array}{c} X_{\mathrm{dsp}}=W_{1}*{\mathrm{Conv}}_{1\times 7}\left(X_{2}\right)+W_{2}*{\mathrm{Conv}}_{7\times 1}\left(X_{2}\right)\\ W_{1}=\mathrm{Sigmoid}\left({\mathrm{Conv}}_{1\times 1}\left({\mathrm{Pool}}_{H\times 1}\left(X_{2}\right)\right)\right)\\ W_{2}=\mathrm{Sigmoid}\left({\mathrm{Conv}}_{1\times 1}\left({\mathrm{Pool}}_{1\times W}\left(X_{2}\right)\right)\right)\\ X_{2}=\mathrm{ReLU}\left({\mathrm{Conv}}_{1\times 1}\left(X\right)\right) \end{array}\right.$$

Equation (6) represents the dynamic strip attention branch corresponding to the lower half of Figure 3. This branch achieves adaptive enhancement of directional features for strip-shaped blurs with inconsistent motion directions. The variable strip attention output $X_{\mathrm{dsp}}$ is obtained by multiplying horizontal and vertical strip features by their respective weights and subsequently summing them, thereby resolving the reconstruction challenge posed by inconsistent motion directions among fragments.

(7) $$X_{\mathrm{ext}}={\mathrm{Conv}}_{1\times 1}\left(\mathrm{Concat}({\mathrm{Conv}}_{3\times 3}\left(X_{\mathrm{pool}}\right),{\mathrm{Conv}}_{3\times 3}\left(X_{\mathrm{dsp}}\right))\right)+X$$

The combined action of multi-scale local features and directional strip features, coupled with residual connections preserving original information, ultimately yields the enhanced feature map $X_{\mathrm{ext}}$. This module possesses adaptive perception and enhancement capabilities for multi-scale, multi-directional non-uniform blurring.

### 2.4. Multi-Scale Feature Fusion Module

**Figure 4:**
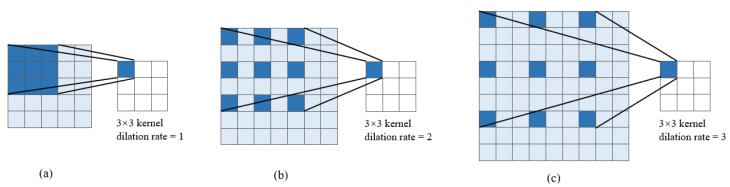
Dilated Convolution: (**a**) dilation rate = 1, (**b**) dilation rate = 2, (**c**) dilation rate = 3.

Dilated convolution achieves the dual objective of expanding the receptive field while preserving the resolution of the output feature map by introducing fixed gaps (dilation) into standard convolution. This approach significantly reduces the loss of image detail caused by downsampling in standard convolution, which is used to increase the receptive field. Dilated convolution introduces the core parameter ’dilation rate’, which defines the pixel spacing between elements within the convolutional kernel. As illustrated in Figure 4: (a) represents standard convolution, which is equivalent to dilated convolution with a dilation rate of 1 and a receptive field of 3×3; (b) depicts dilated convolution with a dilation rate of 2 and a receptive field of 5×5; and (c) depicts dilated convolution with a dilation rate of 3, which yields a receptive field of 7×7. Clearly, a 3×3 dilated convolution achieves the same result as a 5×5 or 7×7 convolution. This approach preserves greater spatial detail by avoiding information loss from direct downsampling and effectively integrates multi-scale feature information.

**Figure 5:**
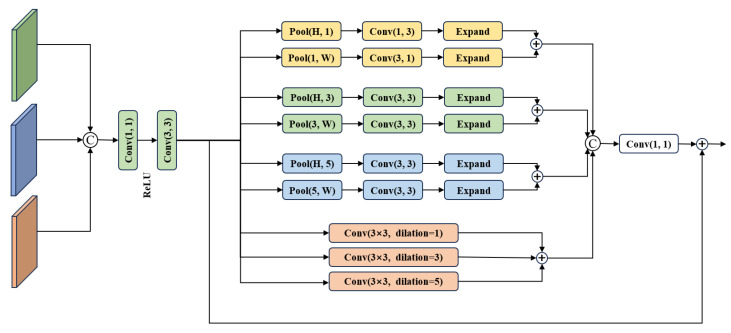
Block diagram of Multi-scale Feature Fusion Module.

During the feature fusion stage, the Multi-scale Feature Fusion Module is designed using dilated convolutions and strip pooling, with its structure shown in Figure 5. This module integrates multi-scale feature maps extracted by the encoder. By employing dilated convolutions, it expands the receptive field while preserving the size of intermediate feature maps, thereby minimizing the loss of spatial detail information. The fused feature maps contain more comprehensive image information, alleviating the limitations of relying on a single feature set. This enhances the robustness of the network’s output, empowering the network to handle images in a wider variety of scenarios.

To effectively restore long-strip motion blur in spatial images, the module employs parallel multi-branch processing to integrate multi-directional and multi-scale contextual information. Adaptive average pooling compresses features along the height and width dimensions, reconstructing feature maps with receptive fields of distinct aspect ratios to capture the structural features of elongated blur in images. Meanwhile, dilated convolutions learn spatial features at various scales. Finally, fusing outputs from multi-directional pooling branches and multi-scale dilated convolution branches significantly enhances the model’s capability to restore elongated blur in spatial images.

The multi-scale features output by the encoder are denoted as $Y_{1}$, $Y_{2}$ and $Y_{3}$, which also serve as inputs to the modules depicted in Figure 5.

(8) $$Y={\mathrm{Conv}}_{3\times 3}\left(\mathrm{ReLU}\left({\mathrm{Conv}}_{1\times 1}\left(\mathrm{Concat}(Y_{1},Y_{2},Y_{3})\right)\right)\right)$$

Equation (8) performs feature fusion on the feature maps from different layers of the encoder, providing a unified input feature for subsequent multi-branch processing.

(9) $$Y_{p1}=\mathrm{expand}\left({\mathrm{Conv}}_{1\times 3}({\mathrm{Pool}}_{H\times 1}\left(Y\right))\right)+\mathrm{expand}\left({\mathrm{Conv}}_{3\times 1}\left({\mathrm{Pool}}_{1\times W}\left(Y\right)\right)\right)$$

(10) $$Y_{p2}=\mathrm{expand}\left({\mathrm{Conv}}_{3\times 3}\left({\mathrm{Pool}}_{H\times 3}\left(Y\right)\right)\right)+\mathrm{expand}\left({\mathrm{Conv}}_{3\times 3}\left({\mathrm{Pool}}_{3\times W}\left(Y\right)\right)\right)$$

(11) $$Y_{p3}=\mathrm{expand}\left({\mathrm{Conv}}_{3\times 3}\left({\mathrm{Pool}}_{H\times 5}\left(Y\right)\right)\right)+\mathrm{expand}\left({\mathrm{Conv}}_{3\times 3}\left({\mathrm{Pool}}_{5\times W}\left(Y\right)\right)\right)$$

Equations (9)–(11) address the diversity in direction and scale of strip-shaped motion blur within images by designing three parallel strip pooling branches. Through adaptive average pooling, these branches form receptive fields of varying scales along both the height and width dimensions, thereby capturing the structural features of strip-shaped blurring.

(12) $$Y_{d}={\mathrm{Conv}}_{3\times 3}^{d=1}\left(Y\right)+{\mathrm{Conv}}_{3\times 3}^{d=3}\left(Y\right)+{\mathrm{Conv}}_{3\times 3}^{d=5}\left(Y\right)$$

Equation (12) designs a multi-scale dilated convolution kernel, achieving the fusion of multi-scale spatial features and addressing the shortcomings of single-scale convolution in perceiving strip-shaped blur patterns.

(13) $$Y_{\mathrm{mffm}}={\mathrm{Conv}}_{1\times 1}\left(\mathrm{Concat}(Y_{p1},Y_{p2},Y_{p3},Y_{d})\right)+Y$$

$Y_{p1}$, $Y_{p2}$, and $Y_{p3}$ represent the results of different pooling operations, respectively, and $Y_{d}$ is the output of convolutions with different dilation rates applied to the feature map. Splicing these together and then incorporating the initial fusion feature map Y via residual connections yields the module output $Y_{\mathrm{mffm}}$. This module can assign higher weights to blurred regions in the image through multi-scale strip pooling and dilated convolution operations, enabling the network to better learn key information in the image and improve the efficiency of feature utilization.

## 3. Results

### 3.1. Datasets and Implementation Details

#### 3.1.1. Dataset

The experimental dataset is generated using star map simulation software. Using the Tycho2 catalog [39], 200 clear point-star-only images are simulated. For each image, five types of motion blur are simulated with blur angles from 0° to 180° and blur lengths from 0 to 90 pixels, yielding 1000 blurred star maps. Specifically, point debris is randomly inserted into the blurred images, while corresponding streak-shaped debris is added to their clear counterparts—thus constructing a simulated dataset with non-uniform motion blur. The dataset is randomly split into 800 training images, 100 validation images, and 100 test images. To mimic real-world image noise, Gaussian noise (mean = 0, standard deviation = 10) is added to the blurred images.

**Figure 6:**
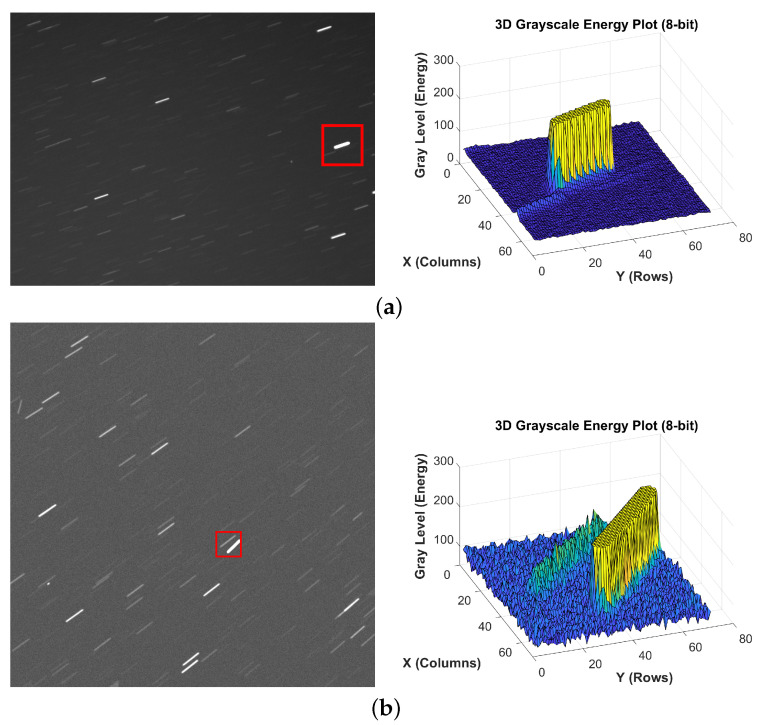
Three-dimensional grey-scale images of elongated trails in real-world and simulated images. (**a**) real-world image. (**b**) simulated image.

As shown in Figure 6, we compared the three-dimensional grey-scale energy distributions of the elongated trails in both the real-world image (spotGEO) and the simulated image. The results demonstrate that the three-dimensional distribution patterns of the two are highly consistent, with the spatial distribution and diffusion range of the core regions closely matching each other. This indicates that the simulated images employed in the experiment exhibit a high degree of correspondence with the actual scenario.

#### 3.1.2. Implementation Details

The entire network framework is implemented using the PyTorch 1.11.0 deep learning framework. The input to the network is 16-bit blurred images with a resolution of 1024×1024. During training, images are randomly cropped to a size of 256×256 pixels. For MSDeblurNet, the loss function combines smooth_L1 and SSIM. The initial learning rate is set to $1\times 10^{-4}$ and gradually reduced to $1\times 10^{-6}$ using a cosine annealing strategy. Additionally, the Adam optimization algorithm is used to iteratively update the network weights, with decay rates $\beta_{1}=0.9$ and $\beta_{2}=0.999$. The training process runs for a total of 1500 epochs, and the experiment is conducted using NVIDIA GeForce RTX 4090D.

### 3.2. Evaluation Metrics

To quantitatively evaluate the image restoration performance of the network, we employ Peak Signal-to-Noise Ratio (PSNR), Structural Similarity Index Measure (SSIM), and centroid error as metrics for quantitative analysis.

PSNR is an objective evaluation metric for image quality pre- and post-processing, defined as the ratio of the energy of the peak signal to the average energy of the noise, with the following calculation formula:

(14) $$\mathrm{MSE}=\frac{1}{mn}\sum_{i=0}^{m-1}\sum_{j=0}^{n-1}{\left[I(i,j)-K(i,j)\right]}^{2}$$

(15) $$\mathrm{PSNR}=10\cdot \log_{10}\left(\frac{{\mathrm{MAX}}_{I}^{2}}{\mathrm{MSE}}\right)=20\cdot \log_{10}\left(\frac{{\mathrm{MAX}}_{I}}{\sqrt{\mathrm{MSE}}}\right)$$

MSE refers to the mean squared error between the restored star image and the clear point star image. I(i,j) denotes the pixel value of the restored star image at position (i,j), and K(i,j) denotes the pixel value of the corresponding clear star image at position (i,j). *m* and *n* represent the number of pixels in the width and height of the image, respectively. ${\mathrm{MAX}}_{I}$ is the maximum possible value of image pixels, which is 65,535 in this experiment.

Structural Similarity Index Measure (SSIM) calculates the similarity by comparing three key features between images: luminance, contrast, and structure [40], which is more consistent with the intuitive perception of the human eye. Its calculation formula is as follows:

(16) $$\mathrm{SSIM}\left(X,Y\right)=\frac{\left(2\mu_{X}\mu_{Y}+C_{1}\right)\left(2\sigma_{XY}+C_{2}\right)}{\left(\mu_{X}^{2}+\mu_{Y}^{2}+C_{1}\right)\left(\sigma_{X}^{2}+\sigma_{Y}^{2}+C_{2}\right)}$$

*X* and *Y* denote the restored image to be evaluated and the clear star image, respectively. $\mu_{X}$ and $\mu_{Y}$ represent the mean values of the two images, while $\sigma_{X}$ and $\sigma_{Y}$ are their standard deviations, and $\sigma_{XY}$ is the covariance. $C_{1}$ and $C_{2}$ are both constants to prevent the denominator from being zero. The closer SSIM is to 1, the higher the structural similarity between the two images.

Centroid Error (CE) represents the deviation distance between the centroid coordinates extracted from the restored star image and the standard centroid coordinates in the clear point-like image. The specific calculation method is as follows:

(17) $$\mathrm{CE}=\frac{1}{N_{\mathrm{image}}}\sum_{i=1}^{N_{\mathrm{image}}}\left[\frac{1}{N_{\mathrm{star}}}\sum_{j=1}^{N_{\mathrm{star}}}\sqrt{{\left(x_{j}-x_{rj}\right)}^{2}+{\left(y_{j}-y_{rj}\right)}^{2}}\right]$$

where $N_{\mathrm{image}}$ is the number of restored star images, $N_{\mathrm{star}}$ is the number of star points extracted from a single star image, $\left(x_{j},y_{j}\right)$ are the standard centroid coordinates, and $\left(x_{rj},y_{rj}\right)$ are the extracted centroid coordinates.

### 3.3. Comparisons with Other Methods

#### 3.3.1. Qualitative Analysis

To verify the effectiveness of the proposed algorithm, comparisons are made with two traditional star image motion deblurring algorithms [24,25] and two deep learning-based motion restoration algorithms [32,41]. In addition, star images with different blur scenarios are selected for testing, specifically: Motion-blurred star images containing blurred faint debris, where the energy of the faint debris is lower than that of nearby debris; Motion blur with overlapping regions, where motion trajectories of multiple objects in the same area are superimposed on each other, resulting in loss of star image details;Non-uniform blurred star images with different blur kernels, where the motion blur lengths and motion blur angles between debris are inconsistent. Since the network is trained on 16-bit unsigned images, the star images and 3D grayscale maps of local regions presented below for visualization have undergone gray-scale stretching.

**Figure 7:**
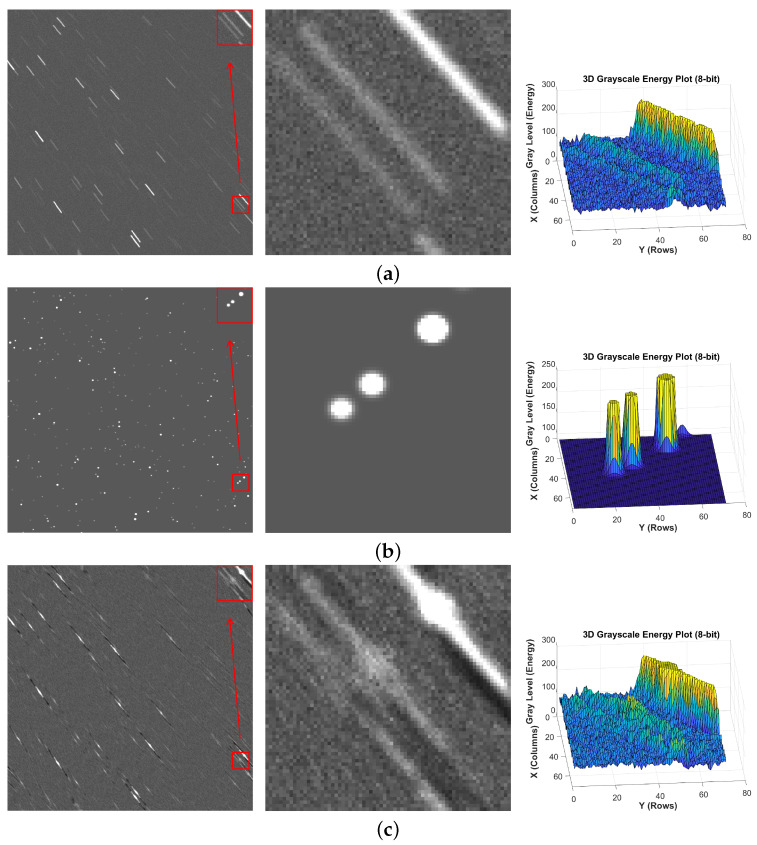

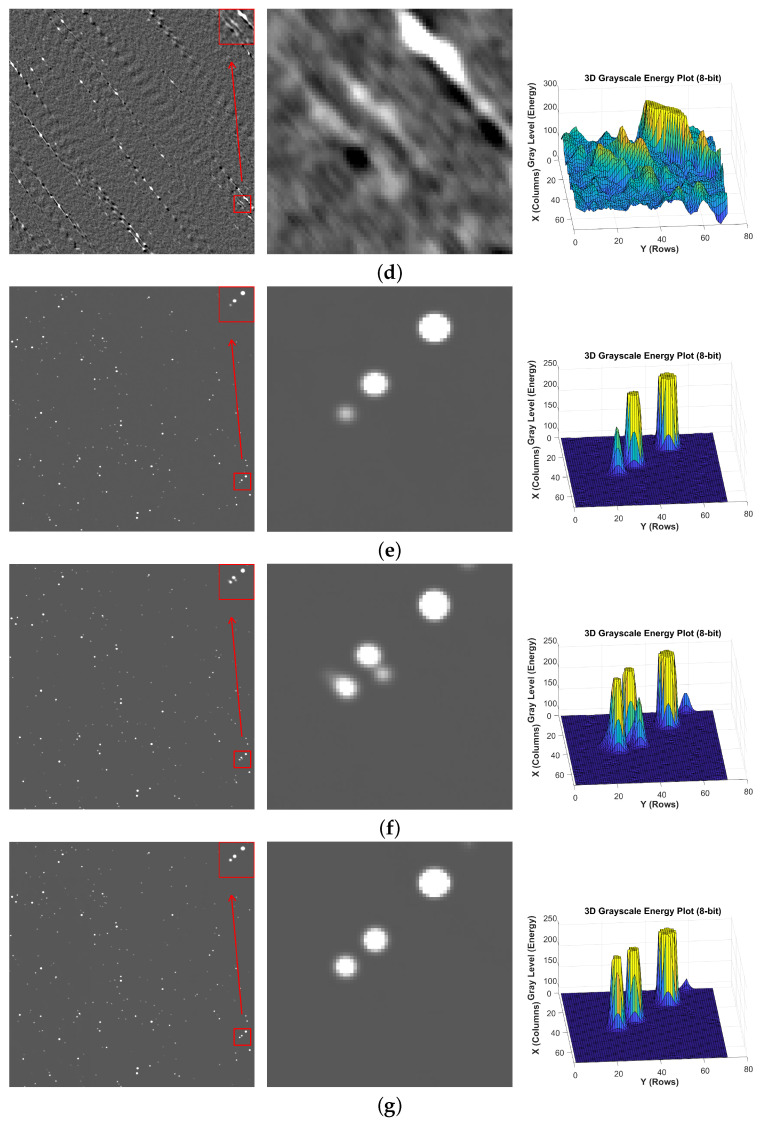
Faint debris: (**a**) blur, (**b**) sharp, (**c**) Method [24], (**d**) Method [25], (**e**) MIMOUNet, (**f**) MIMOUNet_Res, (**g**) MSDeblurNet (star images and 3D grayscale maps).

It can be observed that under noise interference, the strip-shaped faint objects in the blurred star images to be restored are almost submerged in noise. As shown in Figure 7 for faint objects with more dispersed energy after blurring, neither of the two traditional methods can restore the original shape and energy of the debris—especially as the energy decreases significantly under noise influence. Among deep learning methods, MIMOUNet can restore the three debris, but their energy values are lower than those of the debris in the clear star image. MIMOUNet_Res, however, tends to cause over-segmentation (i.e., a single debris is restored as multiple ones). In contrast, the proposed MSDeblurNet in this paper can achieve high-quality restoration of the debris, and their energy can remain consistent with that of the clear debris.

**Figure 8:**
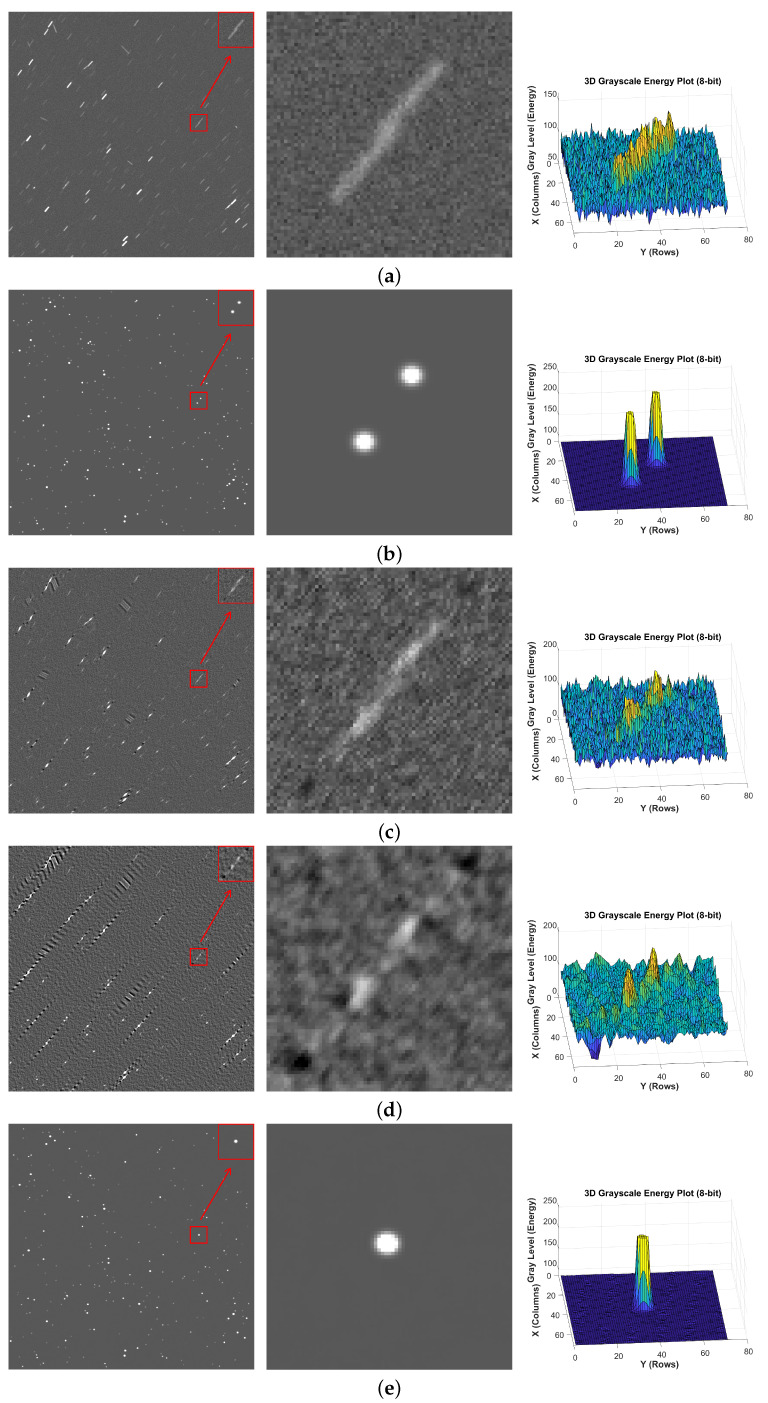

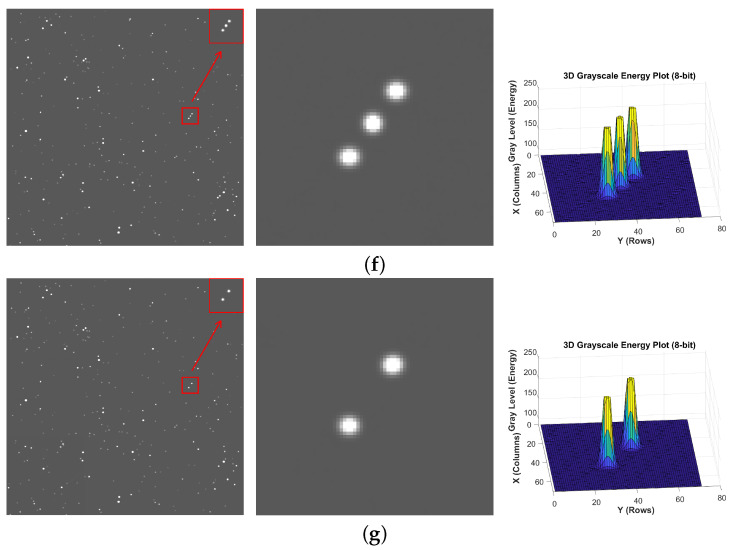
Overlapping debris: (**a**) blur, (**b**) sharp, (**c**) Method [24], (**d**) Method [25], (**e**) MIMOUNet, (**f**) MIMOUNet_Res, (**g**) MSDeblurNet (star images and 3D grayscale maps).

For overlapping debris shown in Figure 8, due to the interference of their blur kernels, image detail information is lost, making it difficult to distinguish the boundaries between debris. The two traditional methods fail in the presence of overlapping, with significant limitations that prevent effective deblurring. Among deep learning methods, MIMOUNet treats overlapping debris as a whole and cannot effectively distinguish overlapping regions. MIMOUNet_Res incorporates a residual learning mechanism, enabling it to capture more details, but it is also prone to incorrect segmentation—restoring two debris as three. In contrast, the proposed method in this paper can well extract and restore two independent debris from the aliased blur kernels.

**Figure 9:**
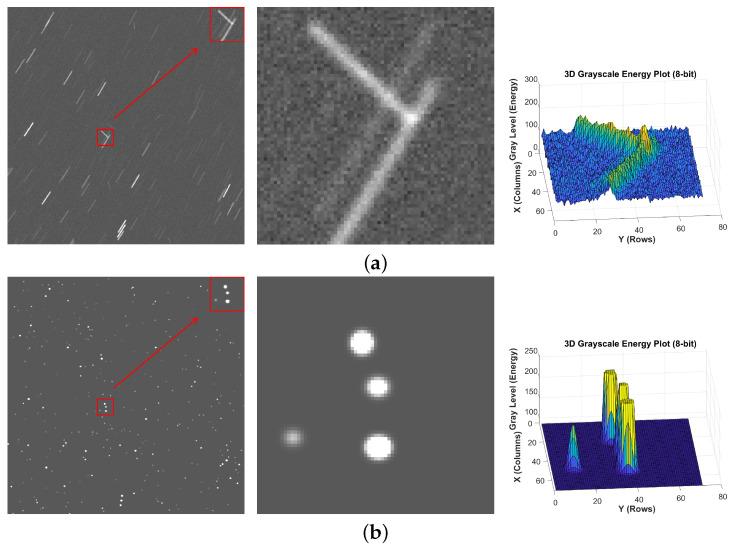

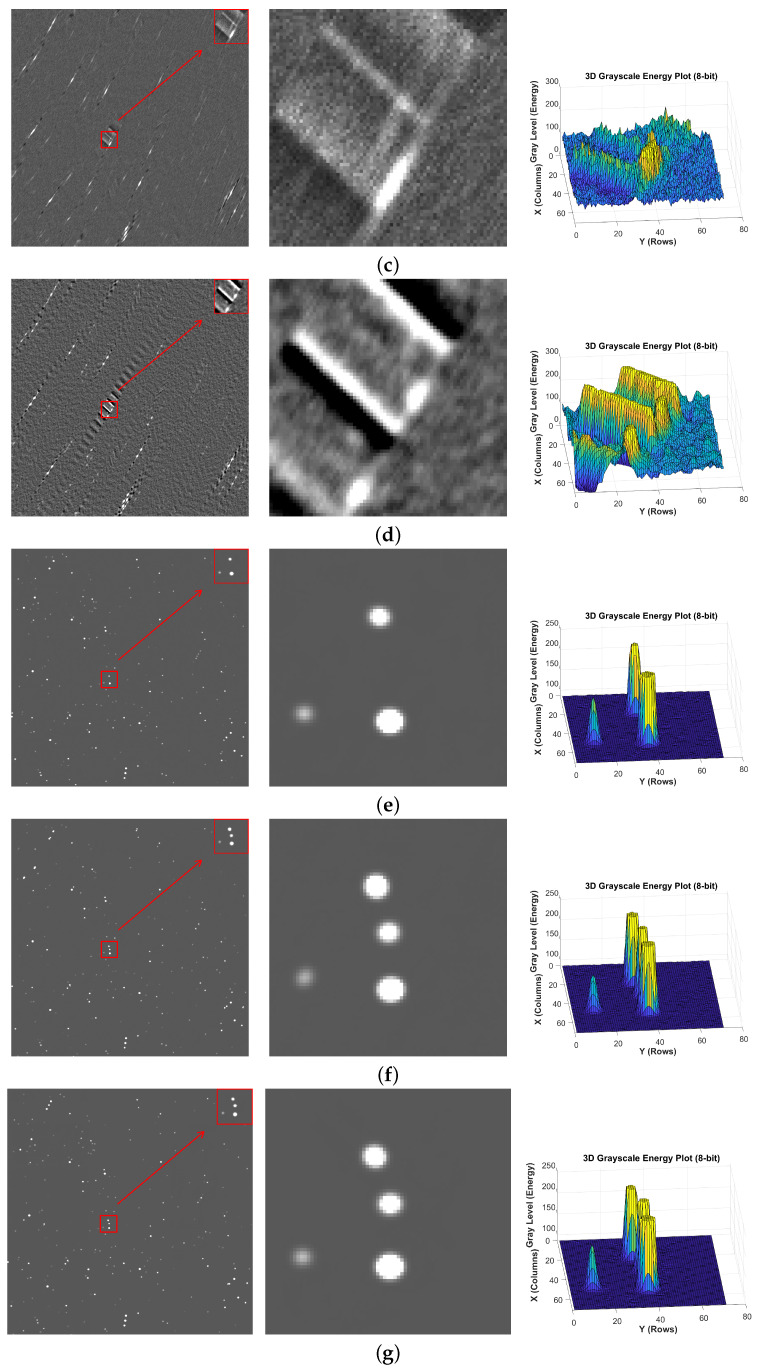
Non-uniform blurred debris: (**a**) blur, (**b**) sharp, (**c**) Method [24], (**d**) Method [25], (**e**) MIMOUNet, (**f**) MIMOUNet_Res, (**g**) MSDeblurNet (star images and 3D grayscale maps).

As show in Figure 9, for the same blurred region with different blur kernels, traditional methods usually assume that image blur follows a single motion blur and can only restore specific blur modes. Thus, when multiple blur scenarios occur in the above figures, the two traditional methods fail to restore effectively. Among deep learning methods, MIMOUNet loses debris information during processing and fails to fully restore the image, while MIMOUNet_Res and MSDeblurNet can achieve image restoration. Specifically, MSDeblurNet performs deeper feature extraction and multi-level feature fusion through MDSPM and MFFM, which significantly improves the accuracy and robustness of restoration.

#### 3.3.2. Quantitative Analysis

Based on the above visual comparison experiments, it can be observed that MSDeblurNet achieves the optimal results for images with faint debris, overlapping debris, and various motion blur scenarios. It can well restore the images and recover the corresponding energy of the debris.

To further quantitatively evaluate the performance of the proposed network, we adopt the evaluation metrics mentioned in Section 3.2: Peak Signal-to-Noise Ratio (PSNR), Structural Similarity Index Measure (SSIM) and Centroid Error(CE) to assess the restored training set data of different restoration algorithms. The results of different algorithms are shown in Table 1:

The table demonstrates that traditional methods have poor restoration results in star image deblurring, whereas deep learning-based restoration achieves PSNR values exceeding 83 dB, with SSIM also showing significant improvement over conventional approaches. Among these, the proposed MSDeblurNet achieves the highest PSNR and SSIM values compared to other comparison algorithms, demonstrating superior capabilities in suppressing detail loss during deblurring and capturing striped features. The designed SDSPM and MFFM effectively restore star images, validating the effectiveness of the proposed methods.

Due to the poor image restoration performance of traditional methods, which hard to recover effective star points, the centroid error analysis compares only deep learning approaches.

As shown in Table 2, all deep learning methods significantly improved the star point extraction rate compared to the original image. Among them, the proposed MSDeblurNet outperformed other comparison models, achieving an extraction rate of 80.37%. Regarding localization accuracy, due to the scarcity of valid samples in the original image, the centroid error holds limited reference value. When comparing among comparable deep learning models, the proposed MSDeblurNet achieved the smallest mean centroid error (0.4589 pixels), demonstrating its optimal star point localization performance.

To validate the computational efficiency of each algorithm, we compared the runtime of different methods on the test set, with results presented in Table 3. The proposed MSDeblurNet algorithm demonstrates significantly superior efficiency compared to traditional methods, processing a single image in 0.3667 s and maintaining comparable computational efficiency to other deep learning–based deblurring methods, completing the entire task in 36.67 s. It mainly relies on algorithms, showcasing an excellent balance between performance and efficiency.

### 3.4. Verification with Real-World Data

To evaluate the practical performance of the proposed MSDeblurNet more comprehensively and objectively, this experiment selected three images from the publicly available spotGEO [42] dataset for supplementary testing. This aims to validate the network’s image restoration capabilities in real-world scenarios. Real-world images exhibit different types of blurring compared to simulated images and feature more complex and diverse backgrounds. Additionally, since real-world images lack corresponding labels for PSNR and SSIM comparisons, this paper evaluates star image restoration performance using star extraction rate and star centroid error metrics.

**Figure 10:**
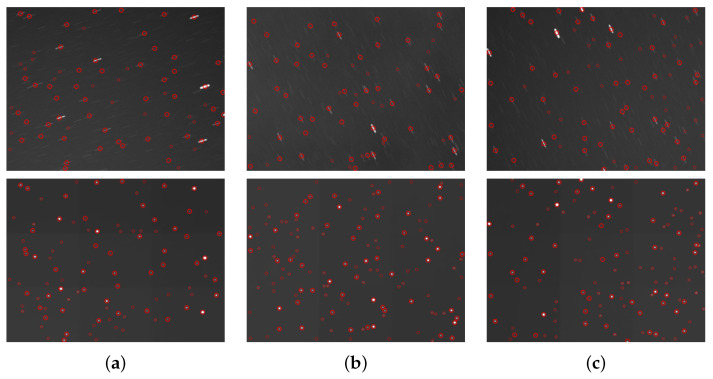
Star point extraction results from the (**a**–**c**) three original star images and the reconstructed star image in the spotGEO dataset. (The red circles indicate the extracted star points.)

Star point extraction is performed using the astronomical image processing software Astrometry.net [43] on both the original measured star image and the restored star image, with results shown in Figure 10. Astrometry.net is an open-source platform for calibrating astronomical images for blind calibration. It integrates geometric hashing techniques, multi-band astronomical catalog data (such as USNO-B and 2MASS), and Bayesian statistical decision methods. Without requiring any prior information—such as image pointing, scale, or orientation—it achieves automatic star map detection, matching, and astrometric calibration (i.e., World Coordinate System WCS information) solely through input images. To quantitatively analyze the extraction results, the number of extracted star points before and after restoration is recorded for comparison. Table 4 presents the specific data on the number of extracted star points:

As shown in Figure 10, the network not only achieves good restoration results for simulated images but also successfully restores the motion blur at different angles from real-world images. Furthermore, Table 4 indicates that the number of extracted star points significantly increases after motion deblurring of real star images, with an average improvement of approximately 57%. The successful restoration and extraction of these star points are crucial for subsequent star image recognition.

To more intuitively analyze the network’s restoration performance for star points, the top ten star points extracted from each original star image are manually annotated to establish a ground truth reference. The coordinates of the restored star points are then compared against this reference to calculate both horizontal and vertical coordinate errors, as well as centroid errors.

**Figure 11:**
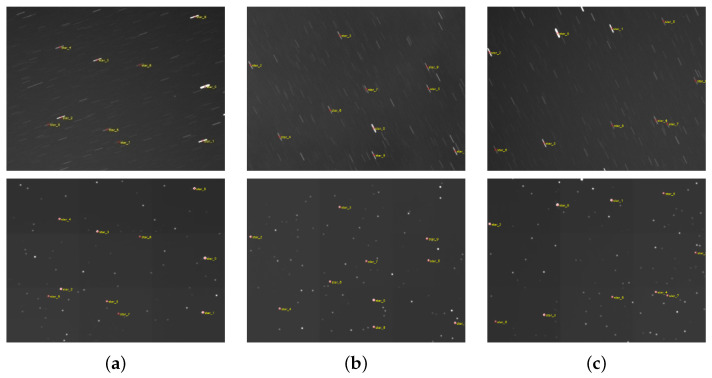
Extract 10 star point result images (**a**–**c**).

The positions of stars in the original image after coordinate correction, compared with those in the restored point star image directly extracted from Astrometry.net, are shown in Figure 11. Table 5 provides the centroid coordinates and centroid errors for 10 stars in the two images depicted in Figure 11b.

The extraction results demonstrate that motion blur-induced star trails in real-world images decrease the number of detectable stars and introduce significant errors in centroid extraction, thus requiring manual recalibration. The proposed MSDeblurNet can effectively restore real-world images while increasing the count of extractable centroids, achieving an average centroid error of 0.456 pixels which is shown in Table 6. However, for real-world images affected by strong stray light, the network yields fewer restored and extracted stars. Such star images necessitate preliminary processing (i.e., background suppression) prior to image restoration.

## 4. Discussion

### 4.1. Ablation Study

#### 4.1.1. Different Crop Sizes Study

To investigate the impact of different image cropped sizes during the training phase on network training effectiveness and restoration performance, this chapter conducts comparative experiments by cropping input images to various dimensions. The results validate model performance across each size, as shown in Table 7.

As shown in Table 7, although the 1024 × 1024 size preserves the complete information of the entire image, its computational load and memory consumption far exceed the capacity of conventional GPUs, preventing the network from completing training. At 512 × 512 resolution, one training epoch pass takes approximately 3300 s—35 times longer than the 256 × 256 resolution used in this study. This extremely low computational efficiency severely impedes model iteration and optimization. The 128 × 128 size offers higher efficiency but yields slightly inferior final restoration metrics (PSNR: 82.23 dB, SSIM: 0.9886). This indicates that the network’s receptive field is insufficient at this size, making it difficult to fully learn long-range motion blur. Furthermore, the cropped images contain too little effective target feature information to support the network in performing high-quality feature learning.

#### 4.1.2. Qualitative Analysis

**Figure 12:**
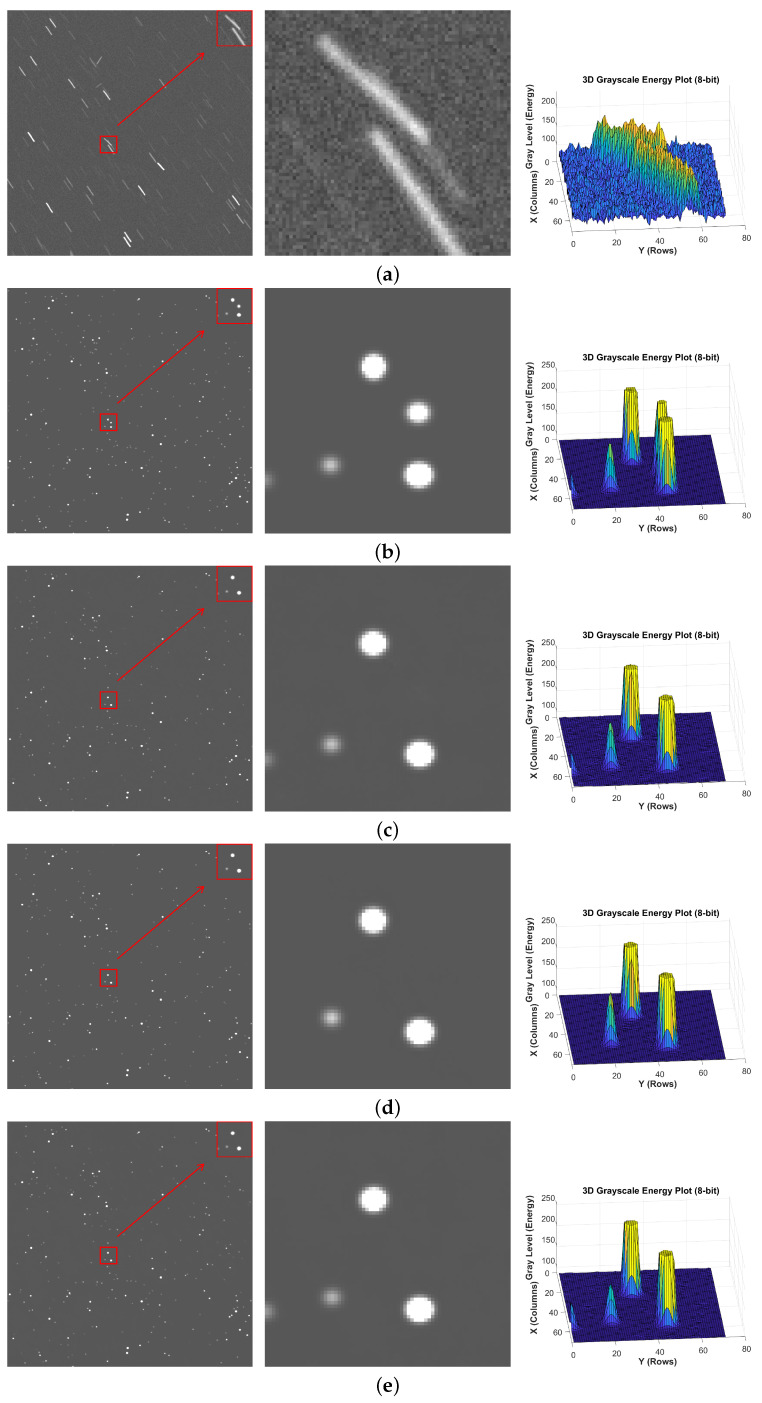

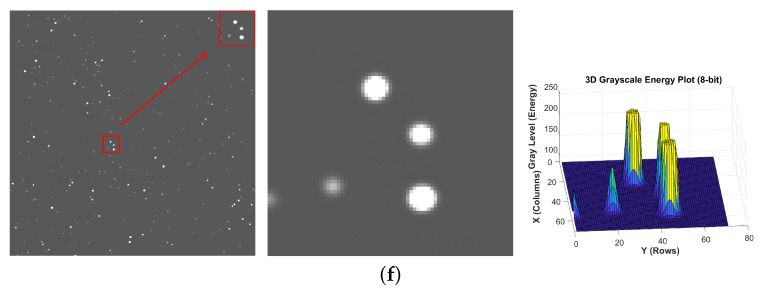
Ablation study results: (**a**) blur, (**b**) sharp, (**c**) Baseline, (**d**) Baseline + MFFM, (**e**) Baseline + MDSPM, (**f**) Baseline + MFFM + MDSPM (star images and 3D grayscale maps.

To further refine the experiments and validate the proposed modules’ enhancement of network deblurring performance, we designed several ablation studies. All ablation experiments employed the same dataset, network training parameters, and experimental platform as Section 3.1. The experiments involved training the base network, a network with the Multi-scale Dynamic Strip Pooling Module added, a network with the Multi-scale Feature Fusion Module added, and a network incorporating both modules simultaneously. The results are shown in Figure 12:

By comparing the restoration performance of different module combinations, it is evident that each combination offers certain advantages in restoring blurred star images. However, in local regions with faint fragments, overlapping fragments, and distinct blurred cores, the Baseline model as well as the MFFM and MDSPM modules—when used independently—deliver suboptimal restoration results. In contrast, when these two modules function synergistically, they enable accurate image deblurring in these complex scenarios.

#### 4.1.3. Quantitative Analysis

Based on the figure above, we can observe the synergistic effect of the two modules in processing complex non-uniform blurred star maps. To further quantitatively validate the specific improvements in model performance achieved by MFFM and MDSPM, we continue to use PSNR and SSIM as evaluation metrics, as mentioned in Section 3.2, to assess models with different module combinations. The specific results are shown in Table 8:

It can be observed from the table that both modules contribute to the improvement of the network’s deblurring performance. Aiming at issues such as detail loss in the extracted feature maps, the MFFM module performs cross-scale fusion of features from different network layers. In contrast, targeting the elongated motion blur in star images, the MDSPM module extracts elongated blur features of varying lengths and directions by introducing a dynamic strip pooling branch, while the square pooling branch is designed to capture global contextual information. Under the synergistic effect of these two modules, the network’s feature learning and feature fusion capabilities are optimized, resulting in a further enhancement of the network performance.

### 4.2. Supplementary Experiments

#### 4.2.1. Different Noise Study

During the training and testing process of the network using simulated datasets, Gaussian noise with a mean of 0 and a standard deviation of 10 was added to the blurred images to simulate the effects of noise. To investigate the image restoration capabilities of the proposed MSDeblurNet under different noise types, multiplicative noise, Poisson noise, and salt-and-pepper noise were respectively introduced to images in the test set. Specifically, multiplicative noise employed a Rayleigh distribution with a scale of 0.1, while salt-and-pepper noise was set to a density of 0.001. Given the salt-and-pepper noise’s pronounced impact on restoration outcomes, we first pre-processed these images using median filtering before applying the proposed MSDeblurNet. For other noise types, MSDeblurNet was applied directly. Experimental results are presented in Table 9, demonstrating that the proposed network achieves favourable restoration performance across diverse noise conditions, exhibiting superiority over alternative methods.

#### 4.2.2. Star Identification

To further validate the practical application value of the proposed deblurring algorithm for subsequent processing, this paper conducts a star identification comparison experiment using actual captured images restored by MSDeblurNet and their original blurred striped counterparts. The investigation aims to determine whether the proposed method can effectively enhance the image processing quality in subsequent applications and improve the final processing results.

**Figure 13:**
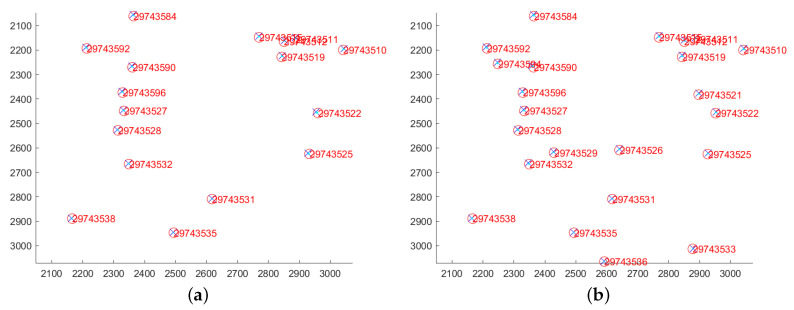
Star identification comparison image: (**a**) Original star point matching results. (**b**) Restored star-point matching results.

We employ the triangular matching algorithm to achieve star map identification, leveraging the geometric relationships between stars for rapid matching. First, a bright star is selected as the origin point, and the angular distances between it and other stars are calculated to form a feature set. This set is then matched against the known data in the Tycho-2 star catalogue, ultimately identifying the information about the stars. The specific experimental results are shown in Figure 13:

Given that the captured image resolution is 6144×6144, direct deblurring of the entire image is constrained by graphics card computational power. Consequently, the original image was cropped into 36 sub-images of 1024×1024 for separate restoration. Figure 12 displays the star point matching results for one such sub-image. The red circles denote the coordinates of known star points from the star catalogue, with the annotated numbers corresponding to the star catalogues. The blue markers indicate the coordinates of star points extracted from the experimental image.

Statistical results indicate that the star catalogue contains a total of 806 stars: the original blurred image identified 712 stellar points, whereas the image restored using the method described herein increased the number of identified stars to 765. This represents a relative improvement in the identification rate of approximately 6.6%, validating that the proposed deblurring algorithm provides benefits for subsequent star map identification tasks.

## 5. Conclusions

This paper proposes an image restoration algorithm for star images with elongated motion blur induced by long exposure times, enabling end-to-end image deblurring. The algorithm adopts an encoder–decoder architecture and employs a multi-scale dynamic strip attention mechanism in the feature extraction stage. Through the design of dynamically weighted strip convolution, it performs differentiated processing on blur patterns, aiding the network in adapting to images with varying blur characteristics. Additionally, addressing the issues that conventional convolutional pooling in the feature fusion stage tends to cause detail loss and insufficient complementarity of cross-level features, this paper innovatively proposes a multi-scale feature fusion module integrating dilated convolution and strip pooling. On one hand, it extracts contextual features via the multi-scale receptive fields of dilated convolution; on the other hand, it captures prominent strip structural information in horizontal and vertical directions through strip pooling. Comparative experiments demonstrate that MSDeblurNet achieves the best performance across all quantitative metrics, including PSNR (84.08 dB), SSIM (0.9928), as well as astronomy-specific star extraction rate (80.37%) and centroid error (0.4589 pixels), with its restoration capability significantly superior to other methods. Further validation on measured data shows that images restored by MSDeblurNet achieve remarkable improvements in the number of extracted stars and centroid positioning accuracy, verifying the proposed algorithm’s strong adaptability to real-world scenarios, along with excellent robustness and effectiveness.

## Figures and Tables

**Table 1:** Quantitative comparison results of different algorithms on the test set.

Method	Method [24]	Method [25]	MIMOUNet	MIMO_Res	MSDeblurNet
PSNR/dB	66.3513	66.8161	83.2999	83.1430	**84.0756**
SSIM	0.2368	0.3166	0.9920	0.9909	**0.9928**

**Table 2:** Centroid error results of different algorithms on the test set.

	Original	MIMOUNet	MIMO_Res	MSDeblurNet
Extraction rate	29.4%	74.57%	76.71%	**80.37**%
Centroid error	0.2910	0.4821	0.5678	0.4589

**Table 3:** Analysis Results of Different Algorithms on the Test Set.

Method	Method [24]	Method [25]	MIMOUNet	MIMO_Res	MSDeblurNet
Total Time/s	453.14	1448.48	29.82	37.41	36.67
Single Image/s	4.53	14.48	0.2982	0.3741	0.3667

**Table 4:** Centroid error results of different algorithms on the test set.

Image	Extracted Stars (Original Image)	Extracted Stars (Restored Image)	Improvement Percentage
1	73	96	31.5%
2	69	135	95.7%
3	84	120	42.9%

**Table 5:** Centroid extraction results of Figure 11b.

Star No.	Centroid		Restoration Centroid		Error		
	X	Y	X	Y	ΔX	ΔY	Centroid Error
star0	371.855	355.267	372.4891	356.4679	0.63407	1.2009	0.581367
star1	610.66	422.824	610.8873	423.3533	0.22727	0.52933	0.499111
star2	10.7866	170.443	11.24112	170.8653	0.45452	0.42225	0.494325
star3	272.179	83.4289	272.3638	83.72476	0.18483	0.29586	0.476343
star4	95.9073	380.701	96.60961	381.3982	0.70231	0.69722	0.4994
star5	532.796	239.834	532.928	240.0887	0.13204	0.25468	0.401521
star6	243.63	302.063	244.1507	302.2044	0.52071	0.14135	0.373729
star7	350.43	241.826	350.683	242.2268	0.25295	0.40081	0.357482
star8	372.106	434.924	372.6101	435.1911	0.50414	0.2671	0.330415
star9	528.288	175.998	528.3717	176.2756	0.0837	0.27763	0.279894

**Table 6:** Centroid Error Results.

Image	1	2	3	Average Error
**Centroid Error**	0.500414	0.429359	0.439496	0.456423

**Table 7:** Performance Comparison of Different Crop Sizes.

Size	Training_TIME/Epoch	PSNR	SSIM
1024 × 1024	–	–	–
512 × 512	3300 s	–	–
256 × 256	93 s	84.0756	0.9928
128 × 128	81 s	82.2307	0.9886

**Table 8:** Analysis results of different module combinations on the test set. (✓ indicates the module is included; - indicates the module is not included.)

Baseline	MFFM	MDSPM	PSNR/dB	SSIM
✓	-	-	83.5803	0.9914
✓	✓	-	83.7634	0.9919
✓	-	✓	83.6343	0.9924
✓	✓	✓	**84.0756**	**0.9928**

**Table 9:** Analysis Results of Different Algorithms on Test Sets with Different Noises.

Data		Met. [24]	Met. [25]	MIMOUNet	MIMO_Res	MSDeblurNet
Multiplicative	PSNR/dB	66.85	66.84	74.76	73.90	74.17
	SSIM	0.2503	0.5207	0.9614	0.9870	0.9875
Poisson	PSNR/dB	66.99	67.08	83.24	83.03	83.93
	SSIM	0.4467	0.3388	0.9919	0.9913	0.9930
Salt_Pepper	PSNR/dB	68.40	67.31	83.06	82.81	84.10
	SSIM	0.9315	0.8862	0.9940	0.9955	0.9946

## Data Availability

The original contributions presented in this study are included in the article. Further inquiries can be directed to the corresponding author.
